# Correlation between the estimated GFR and SWRD score in patients with posterior urethral valves at King Abdul-Aziz University Hospital

**DOI:** 10.1186/s13104-019-4120-8

**Published:** 2019-02-12

**Authors:** Afnan Neyas, Rana Bajaba, Rahaf AlThomali, Rahaf Alturkistani, Baraah AlSawaf, Weaam Alrefai, Lujain Hefni, Lamees Aldoobie, Sherif Desoky, Jameela Kari, Osama Y. Safdar

**Affiliations:** 1 Faculty of Medicine & Surgery, King Abdulaziz University, Jeddah, Saudi Arabia; 2 Pediatirc Nephrology Center of Excellence, King Abdulaziz University, Jeddah, Saudi Arabia

**Keywords:** Posterior urethral valves, SWRD, PUV, GFR

## Abstract

**Objective:**

The aim was to establish the reliability of the SWRD score as a predictor of both renal and bladder outcomes in posterior urethral valves. This retrospective study included 67 patients with PUVs at King Abdul-Aziz University Hospital. The score was calculated from voiding cystourethrogram before and after the relief of obstruction, and estimated glomerular filtration rates (eGFRs) were calculated as well.

**Results:**

Based on Spearman correlations, both baseline eGFRs and SWRD scores can be possible predictors of long-term renal outcomes, as a significant positive correlation between the baseline eGFRs and the last eGFRs was found (p = 0.005). A significant negative correlation was also found between the SWRD score calculated before the intervention and the last eGFRs (p = 0.02). Additionally, the baseline SWRD scores can be possible predictors of short-term bladder outcomes, as the correlation analysis showed a positive relationship between the baseline SWRD scores and the SWRD scores calculated within 2 months after the intervention (p < 0.0001). A significant decrease in SWRD scores and eGFRs was found from before to after the intervention, regardless of the type of intervention. In conclusion, the SWRD scoring system proved to be a potentially promising tool in the anticipation of the clinical outcomes of PUVs.

## Introduction

Posterior urethral valves (PUVs) are well known for their unique sequelae of causing bladder dysfunction after the relief of obstruction, and for this reason in particular, chronic kidney disease (CKD) commonly develops [1]. Although the incidence of PUVs is approximately 1 in 5000 [2], this condition accounts for end-stage renal disease (ESRD) in a substantial proportion of children [3].

The disease has a relatively wide spectrum of presentations depending on its severity and type, and given the widespread use of antenatal ultrasonography, the prenatal diagnosis of PUVs is becoming increasingly common [4]. The diagnosis is usually confirmed postnatally through voiding cystourethrogram (VCUG) showing the classical findings of PUVs [5].

Many previous studies have identified gaps in the research on the predictors of general long-term renal or bladder-related clinical outcomes in patients with PUVs [1, 6]. Nonetheless, several parameters have been suggested to predict renal outcomes in these patients. A study was conducted in our hospital with nearly the same population to investigate the predictors of renal impairment [7]. The study showed a statistically significant difference in the duration of follow-up, nadir serum creatinine, last known serum creatinine level, presence of VUR, and baseline and final estimated glomerular filtration rate (eGFR) between the patients with and without renal impairment in the sample.

A recent study proposed the SWRD scoring system, which stands for the shape of the bladder, trabeculations of the bladder wall, presence of reflux, and presence of bladder diverticula when the bladder was at two-thirds of its maximum capacity; this scoring system reliably quantifies the cystometrogram (CMG) appearance of the bladder in PUV patients [8]. It was essentially developed to address the problem of inconsistent reports due to the subjective assessment of imaging modalities, which can be used as a prognostic indicator of the need for intervention and can offer objective and early predictions of bladder outcomes.

It was also reported that the SWRD score can be obtained from VCUG imaging alone, reducing the need for CMG studies; our current study was based upon this premise, due to the lack of CMG studies in our sample. Thus, our study aimed to investigate the correlation between the SWRD score, which represents the bladder outcomes, and the estimated glomerular filtration rate (eGFR), which represents the renal outcomes, in patients with PUVs. It was anticipated that this additional study might contribute to the literature regarding the reliability and potential use of the SWRD score for bladder outcomes as a surrogate for renal outcomes in these patients.

## Main text

### Study design and population

This study was a cohort retrospective record review that included all patients admitted to the paediatrics ward in King Abdul-Aziz University Hospital with a diagnosis of PUVs or patients admitted and diagnosed during their stay between 2001 and 2018.

### Instruments and Biochemical measurements

The diagnosis was confirmed by voiding cystourethrogram (VCUG) and cystoscopy. All patients whose records had a significant amount of missing data or, those who had a miscellaneous diagnosis were excluded.

The SWRD scoring system’s scheme was built to give a minimum score of 0 for radiographically normal bladders and a maximum score of 7 for the most radiographically abnormal bladders. In this research, the scores were obtained from the pre- and post-intervention VCUG images, and they were calculated by 2 blinded assessors. Post-intervention VCUGs were usually performed 1–2 months after the last intervention and served as our 2nd calculated score; they would then be performed again at another visit, either as a follow-up or due to recurrent symptoms, and this score would be considered the 3rd/last calculated score. The interval between both post-intervention scores varied among patients, but the minimum duration was identified to be at least 2 years.

Moreover, the patient’s pre-intervention serum creatinine level and last creatinine in his previous follow-up had been used to predict renal dysfunction by the calculation of the eGFR using the Schwartz formula [CrCl (ml/min) = K × height in cm/scr (mg/dl)], where K = 0.45 for infants, k = 0.55 for children less than 18 years old and K = 0.7 for males > 18 years old.

### Statistical analysis

The collected data were verified, coded, entered into an owned computer, and analysed in the Statistical Package for Social Sciences (SPSS) program v21. Data were presented as numbers and frequencies or as medians (quartile 25–75). Spearman correlations were used. Statistical significance was set at P < 0.05.

### Results

Out of 67 patients, 42 (62.7%) were Saudi. Almost half of the cases (32 cases; 47.8%) presented during the period of 2006–2011.

Regarding the type of intervention, fulguration was performed in 41 (61.2%) cases, followed by vesicostomy in 24 (35.8%) cases, and both procedures -vesicostomy and then fulguration-were performed in 2 cases, with a median follow-up duration of 3 years.

The use of clean intermittent catheterization (CIC) was indicated in 9 (13.4%) cases, and 26 patients (38.8%) had hypertension.

The median values for several variables are listed as follows: 4 for the 1st calculated SWRD score (calculated from VCUGs performed just before the intervention), 29.1 for the 1st eGFR (calculated from creatinine taken just before intervention), 3 for the 2nd SWRD score (calculated from VCUGs performed within 2 months after the last intervention), 49.9 for the 2nd eGFR (calculated from creatinine taken in the last follow-up visit), and 2 for the last SWRD score (calculated from VCUGs performed with a minimal duration of 2 years after the last intervention) (Table 1).

**Table 1:** Scores

Variables	Median	Quartile (25–75)
Calculated SWRD score before the intervention (1st score)	4	3.0–5.0
Estimated GFR before the intervention (1st eGFR)	29.1	15.2–45.1
Calculated SWRD score within 2 months after the last intervention (2nd score)	3	2.0–4.5
Estimated GFR in the last follow up visit (2nd eGFR)	49.9	17.7–108.1
Calculated SWRD score after a minimal duration of 2 years after the last intervention (last score)	2	1.8–3.3

The results in Table 2 reveal a significant decrease in the SWRD scores when they were compared before and immediately (i.e., within 2 months) after the intervention, from 4 to 3, regardless of the type of intervention; furthermore, a larger difference between the scores was found (from 4 to 2) when calculated before and after the intervention with a minimal duration of 2 years, regardless of the type of intervention (p = 0.005). In addition, a significant increase was found in the eGFR taken before and after the intervention, from 29.1 to 49.9 (p = 0.0001).

**Table 2:** Comparing scores and measurements

Variables	Median of the scores before intervention	Median of the scores after the intervention	p value
SWRD	4	3	0.005
eGFR	29.1	49.9	0.0001

Upon dividing the sample into two intervention groups, namely, vesicostomy and fulguration, and comparing the 2nd SWRD score (short-term outcome) with the last SWRD score (long-term outcome), a slight improvement in the scores was found (2.7 vs. 2.0, p = 0.32 in the fulguration group and 3.0 vs. 2.5, p = 0.15 in the vesicostomy group) (Table 3).

**Table 3:** Comparing scores after intervention (short-term vs. long-term outcome)

Variables	2nd SWRD score	Last SWRD score	p value
Vesicostomy	2.7	2.0	0.32
Fulguration	3.0	2.5	0.15

Spearman correlations showed a negative correlation between the 1st SWRD and the 2nd eGFR (r = − 0.358, p = 0.02) and a positive correlation between the 1st eGFR and the 2nd eGFR (r = 0.391, p = 0.005). Additionally, there was a positive correlation between the 1st SWRD and the 2nd SWRD (r = 0.703, p < 0.0001). A negative correlation was found between the 1st SWRD and the 1st eGFR (r = − 0.220, p = 0.18).

### Discussion

To our knowledge, this is the 2nd study that investigates bladder outcomes using SWRD scores in PUV patients after the emerging study; thus, there were not enough references regarding the reliability of the score. However, in our study, we attempted to find a correlation between renal outcomes using the scores calculated before the intervention for both the estimated GFRs and bladder outcomes. A negative correlation between the parameters was found, which is expected given that the higher the score is, the worse the bladder outcome, and the lower the eGFR is, the worse the renal outcome. Bladder dysfunction before the intervention can be attributed to the antenatal nature of the disease and its chronic obstructive effect.

Based on the correlations found in this study, both baseline eGFRs and SWRD scores can be possible predictors of long-term renal outcomes, meaning that the significant positive correlation between the baseline eGFR and the last eGFR indicates that renal function at the time of presentation can be a predictor in the long term. The significant negative correlation that was found between the baseline SWRD score and the last eGFR showed that it can also be used as a predictor; the higher the score is at the time of presentation of a PUV patient, the lower the score and, consequently, the worse the renal outcome, even after the intervention.

Furthermore, the significant positive correlation between the baseline SWRD score and the score calculated within 2 months after the intervention indicates that baseline scores can be used as predictors of bladder outcomes.

Long-term renal and bladder outcomes are vital determinants of quality of life in PUV patients [6]. Unfortunately, even after successful relief of obstruction, patients may still have various urological issues related to disease severity at the time of presentation [9], which raises the question of the extent to which bladder outcomes can be improved after the intervention. Our study showed an improvement in the median of the scores after the intervention, both for the short-term outcomes (from 4 to 3) and for the long-term outcomes (from 4 to 2). It was reported that early detection and reversal of bladder dysfunction are associated with a better chance of preservation of kidney function [9, 10], and improvement in the degree of bladder dysfunction and, thus, the associated scores might be associated with better kidney outcomes, lower rates of infection, lower severity of neurogenic bladder and less-frequent daily use of CIC.

A predilection was shown for fulguration in terms of long-term bladder outcomes, as the differences between the 2nd SWRD score within 2 months after the intervention and the last score calculated with a minimum duration of 2 months were 0.5 and 0.7 for the fulguration and vesicostomy groups, respectively; however, this difference was statistically insignificant. The preferable method of intervention that is associated with better outcomes is still a matter of debate, although the risk of bladder dysfunction and disturbed urodynamic parameters is increased upon diversion [11–13]. However, some papers have reported better bladder outcomes in some cases [14–16], and others were in favour of ablation [13, 17].

Vesicostomy should be reserved mainly for very-low-birth-weight infants and patients with continued impaired renal function, upper tract deterioration after valve ablation and late presentation [18–20]. However, in our population, the use of clean intermittent catheterization (CIC) post-fulguration was not the preferred solution for many individuals, as we had faced major cultural rejection; therefore, vesicostomy was an appropriate temporary solution in many selected cases.

CIC is an important asset in managing children with noncompliant bladders [8]. However, in this context, the inability or unwillingness to adhere to the catheterization time schedule and follow-ups by the parents or the children themselves represented a great challenge, and concerns about long-term compliance with CIC resulted in a higher rate of vesicostomy in comparison to other samples.

Furthermore, the children’s and parents’ fears of potential pain and harm to body parts [21], the possible complications, including strictures, and the inadequacy of our educational and supportive system [19] all contributed to the choice of having vesicostomy as a primary temporary solution in a portion of our cases.

The direct comparison of our results to those of Niyogi et al. [8] is not feasible, as they calculated only one score after the primary intervention when VUD was indicated either for symptoms or as part of routine surveillance at 5–15 years of age. The median score for patients who required intervention was 2, which was lower than the median score for all our patients before their first primary intervention. Thus, this score might not be objective because the scores were calculated for patients after a previous primary intervention, which might have led to a relative improvement in bladder function.

In conclusion, the SWRD scoring system proved to be a potentially promising tool in the anticipation of the clinical outcomes of PUVs. SWRD scores can also be used as an assessment tool for future interventions.

## Limitations

This study had three main limitations. The retrospective nature of the study required us to address the problem of missing data, there were inconsistent reports of VCUGs, and the study sample was relatively small and was taken from a single healthcare center. A potential limitation of the score is the nature of the vesicostomy procedure, as two points are given for a grossly distorted bladder, which may overrate the post-intervention scores and bladder outcomes in this group of patients.

## Abbreviations

PUV: posterior urethral valves
GFR: glomerular filtration rate
eGFR: estimated glomerular filtration rate
ESRD: end-stage renal disease
VUR: vesicoureteric reflux
CIC: clean intermittent catheterization
VCUG: voiding cystourethrogram
CMG: cystometrogram
CKD: chronic kidney disease
